# Limitations of CT scanning in Bosniak staging of renal cystic carcinoma

**DOI:** 10.1093/jscr/rjy052

**Published:** 2018-04-12

**Authors:** A S Wasim, F Mumtaz

**Affiliations:** 1 University Hospital Coventry, Core Surgery, Coventry CV2 2DX, UK; 2 Royal Free London NHS Foundation Trust, Urology, London NW3 2QG, UK

## Abstract

The Bosniak Classification is used to quantify the risk of malignancy and need for observation or radical treatment based on the findings of computed tomography (CT). The case described is that of a 65-year-old man with renal cystic disease who was initially given a Bosniak stage IIF classification and was subsequently managed with CT surveillance. CT surveillance showed increased cyst size in the left kidney with cystic changes, however, the Bosniak classification remained the same. It was not until the patient deteriorated further that an MRI was indicated. The MRI findings upgraded the lesion from Bosniak IIF to Bosniak III. As a result, the patient underwent a radical left nephrectomy and a biopsy, which revealed clear cell carcinoma. This case argues the limitations of the Bosniak classification and the value of using MRI at an earlier stage, especially with unusual circumstances such as a chronic history of enlarging cysts.

## INTRODUCTION

The 50% of the UK population over 50 years old will have at least one simple renal cyst (SRC). However, a small number of these patients may have advanced renal cystic disease, such as a complicated renal cyst (CRC) or development of a malignant renal cyst [1]. The risk of malignancy can be quantified using the Bosniak classification based on the findings of computed tomography (CT) [2, 3]. CT scanning has been the gold standard diagnostic intervention utilized with regards to renal cystic disease and is also used as a surveillance tool [4]. Magnetic resonance imaging (MRI) may give greater anatomical detail of a renal lesion. However, it is usually reserved for those who have been diagnosed with complicated cysts with malignant potential on CT, individuals who would not tolerate CT, or patients with clinical symptoms [5]. As a result, it is possible for a patient to be on longstanding CT surveillance with the diagnosis of SRC with low malignant potential whereas an MRI would have upgraded the lesion to a higher Bosniak stage.

## CASE PRESENTATION

A 65-year-old male was referred to the urology clinic in 2012 for suspected renal cystic disease discovered incidentally on CT. There were no significant findings on examination and the patient was asymptomatic.

### Investigations

A contrast enhanced CT noted small SRCs bilaterally, the largest being 1.2 cm in width. A 10.5 cm upper pole simple cyst was also noted in the left kidney. It contained a small soft tissue focus in the lateral wall, however, no calcification, septations or convincing solid components were noted. The lesion fit the criteria for Bosniak stage IIF and the patient was placed on 12-month interval CT surveillance.

In 2013, significant increased global cyst size was noted in the left kidney, the largest being a 7.5 cm lesion in the left lower pole with a small focus of wall calcification. The left kidney upper pole cyst had increased in size to 10.7 cm with new focal soft tissue calcification noted. However, these findings were still consistent with Bosniak stage IIF. A decision was made to alter CT surveillance from 12- to 6-month intervals.

**Figure 1:**
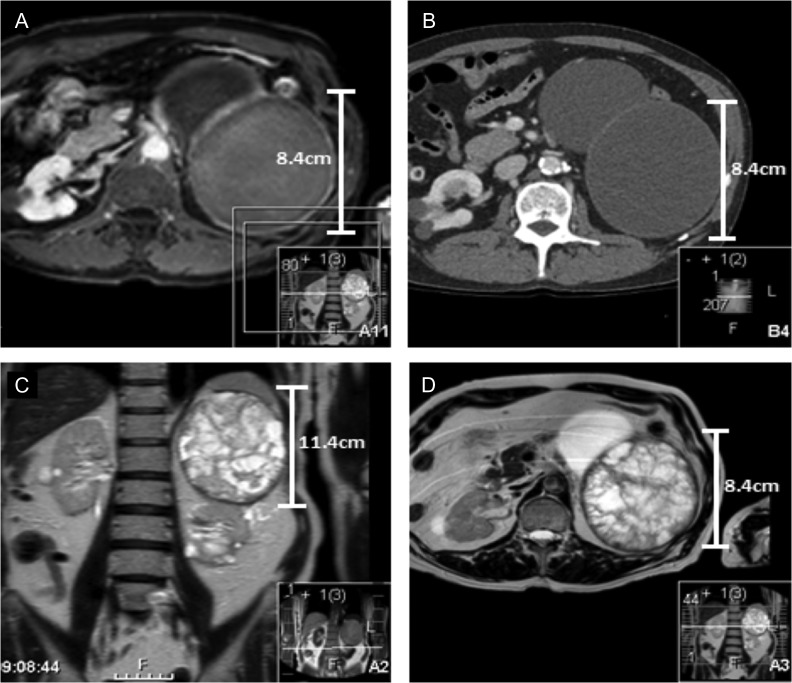
(**A** and **B**) Represent contrast and non-contrast enhanced CT scans from 2014 of the left upper pole cyst when it was noted that the patient complained of 6 months of weight loss. The CT findings indicated the upper pole cyst to still be classified as Bosniak stage IIF. There is no notable calcification, septations or convincing solid components. (**C** and **D**) Both represent the MRI of the upper pole cyst. The findings images demonstrate extensive, thick septations in the upper pole cyst upgrading the cyst to Bosniak stage III. The scans outline the limitations of surveillance CT vs MRI to stage the severity of renal cystic disease in this patient.

In 2014, the lower pole cyst in the left kidney had increased in size from 7.5 to 9.4 cm. A further 9.3 cm cyst had developed from the anterior midpole. The upper pole cyst had increased in size from 10.7 to 11.4 cm. The radiographic findings still indicated these cysts to be simple in nature. At this time the patient reported weight loss of 5 kg and fatigue over the last 6 months. eGFR had deteriorated to 29 ml/min. MRI corroborated CT results at this stage. MRI also demonstrated the left renal upper pole cyst to contain extensive thick walled septations. T1 gadolinium imaging demonstrated significant underlying haemorrhage. MRI findings upgraded the lesion from Bosniak IIF based on CT findings to Bosniak III. MRI and CT images illustrating these findings are outlined in Fig. 1.

### Treatment

The patient underwent an open radical left nephrectomy and a biopsy revealed clear cell carcinoma (Fuhrman grade 2). Resection margins were clear and overall the findings indicated good prognosis despite the large tumour size. Routine follow-up revealed no significant symptoms or complications. CT and MRI scanning indicated no sign of further complex renal cystic disease.

## DISCUSSION

The majority of renal cysts are SRCs and generally do not require treatment [5, 6]. However, CRCs can develop from SCRs or independently. Unlike SRCs, CRCs carry an increased risk of malignancy [1]. Therefore, accurate differentiation between the two lesions is crucial.

**Figure 2:**
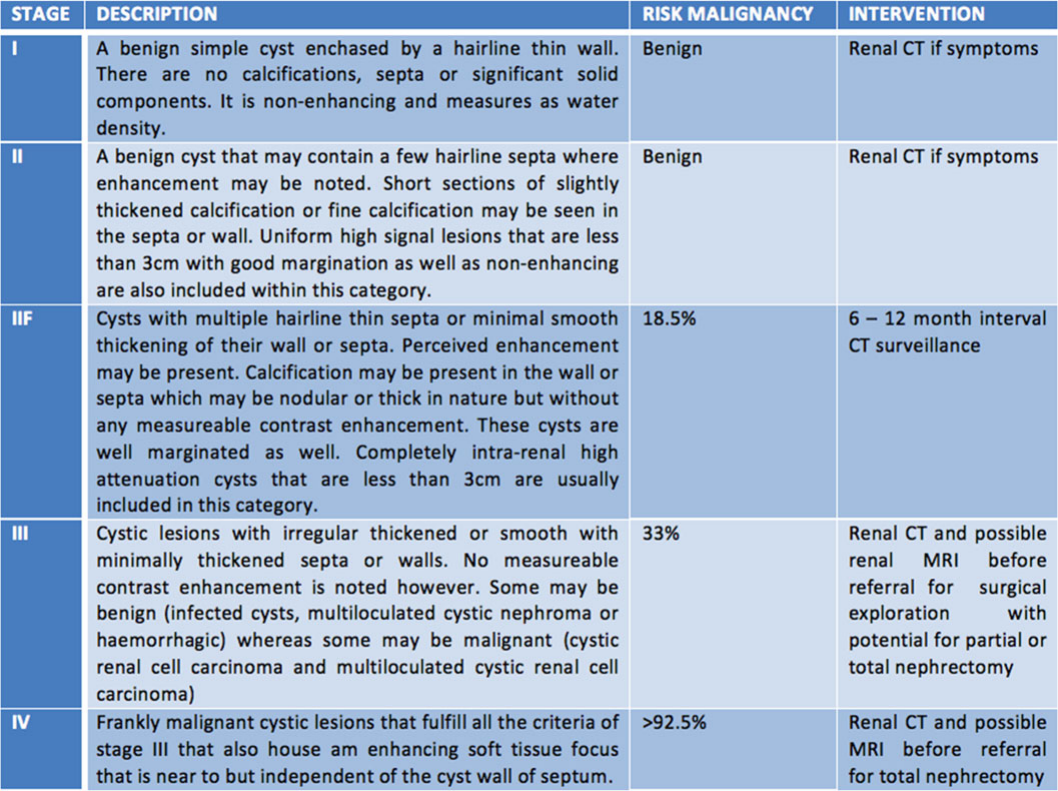
Bosniak classification of renal cystic lesions [4].

Bosniak staging governs the decision to operate on CRCs [2, 3]. This classification of renal cystic lesions was produced in an attempt to standardize the evaluation of the condition across all imaging modalities. It quantifies the risk of malignancy and the need for observation or radical treatment [6]. The full classification and implicated treatment regimen is outlined in Fig. 2.

CT scanning is the gold standard diagnostic intervention in renal cystic disease, with MRI reserved as a second line modality. However, MRI findings can also be correlated to the classification due to its ability to identify similar characteristics as CT scans [3]. There are also scenarios where MRI is superior to CT in determining cystic disease as it can confirm septations and parietal thickening that can be missed on CT [7]. In a retrospective analysis of CT and MRI from 68 renal masses, MRI findings corresponded with CT staging in 81% (56). Of the 19% where MRI did not match CT staging, 10% (seven cases) of the lesions were upgraded to higher Bosniak staging due to MRI findings of increased septations and pronounced septal and or wall thickness [8].

The patient in the scenario had been on longstanding CT surveillance which exhibited growing SRCs and low grade CRCs. It is common for patients with renal cystic disease to remain asymptomatic as was the case with this patient. In the event that this patient had not developed clinical symptoms, an MRI may not have been indicated [9]. This case suggests the value of using MRI at Bosniak IIF especially with unusual circumstances such as a chronic history of enlarging cysts. A recent cohort study assessing transformation of SRCs supports this reasoning. Cases of renal cell carcinoma derived from SRCs had their medical records analysed retrospectively. In total, 17 cases had been diagnosed via CT and MRI as having SRC for over 6 months. Surgical decortication was opted for in these cases upon onset of complicated variations of SRC. Histopathological analysis revealed all 17 as clear cell renal carcinoma (Fuhrman grades I–III) [10]. The study indicates that despite scans outlining no evidence of complex renal cystic disease, unusual disease progression could indicate the onset of malignancy. This case demonstrates the value in being cautious with patients who do not follow the stereotypical progression of SRCs.

## CONCLUSION

It is important to remember CT scanning can falsely stage renal cysts lower than MRI where septations are present in the lesion. In patients with complicated simple renal cystic disease, MRI should be considered early to aid accurate Bosniak classification.
